# Kinematics of fast cervical rotations in persons with chronic neck pain: a cross-sectional and reliability study

**DOI:** 10.1186/1471-2474-11-222

**Published:** 2010-09-27

**Authors:** Ulrik Röijezon, Mats Djupsjöbacka, Martin Björklund, Charlotte Häger-Ross, Helena Grip, Dario G Liebermann

**Affiliations:** 1 Centre for Musculoskeletal Research, University of Gävle, Sweden; 2 Department of Community Medicine and Rehabilitation, Physiotherapy, Umeå University, Sweden; 3 Alfta Research Foundation, Alfta, Sweden; 4 Department of Biomedical Engineering & Informatics, University Hospital of Umeå, Umeå, Sweden; 5 Department of Physical Therapy, Sackler Faculty of Medicine, Tel Aviv University, Israel

## Abstract

**Background:**

Assessment of sensorimotor function is useful for classification and treatment evaluation of neck pain disorders. Several studies have investigated various aspects of cervical motor functions. Most of these have involved slow or self-paced movements, while few have investigated fast cervical movements. Moreover, the reliability of assessment of fast cervical axial rotation has, to our knowledge, not been evaluated before.

**Methods:**

Cervical kinematics was assessed during fast axial head rotations in 118 women with chronic nonspecific neck pain (NS) and compared to 49 healthy controls (CON). The relationship between cervical kinematics and symptoms, self-rated functioning and fear of movement was evaluated in the NS group. A sub-sample of 16 NS and 16 CON was re-tested after one week to assess the reliability of kinematic variables. Six cervical kinematic variables were calculated: peak speed, range of movement, conjunct movements and three variables related to the shape of the speed profile.

**Results:**

Together, peak speed and conjunct movements had a sensitivity of 76% and a specificity of 78% in discriminating between NS and CON, of which the major part could be attributed to peak speed (NS: 226 ± 88 °/s and CON: 348 ± 92 °/s, p < 0.01). Peak speed was slower in NS compared to healthy controls and even slower in NS with comorbidity of low-back pain. Associations were found between reduced peak speed and self-rated difficulties with running, performing head movements, car driving, sleeping and pain. Peak speed showed reasonably high reliability, while the reliability for conjunct movements was poor.

**Conclusions:**

Peak speed of fast cervical axial rotations is reduced in people with chronic neck pain, and even further reduced in subjects with concomitant low back pain. Fast cervical rotation test seems to be a reliable and valid tool for assessment of neck pain disorders on group level, while a rather large between subject variation and overlap between groups calls for caution in the interpretation of individual assessments.

## Background

The pathophysiology behind chronic neck pain remains largely unresolved. Specific diagnoses are rare, and a majority of neck pain disorders are considered non-specific. Important improvements in the characterization of neck pain conditions have been attained from research on sensorimotor functions. Altered sensorimotor functions in neck pain disorders include reduced range of movement (ROM) [1-3], reduced proprioceptive sensibility [4-6], altered activation patterns of cervical muscles [7], reduced maximal speed [8,9] and movement smoothness [6,8,10] during cervical movements.

A majority of the studies that characterized cervical movements in people with neck pain have involved slow or self-paced speed tasks. Functional head movements in daily living may also entail fast rotations. For instance, rapid changes of gaze direction require fast cervical rotations if the magnitude of gaze change is large. Two reports on fast cervical rotations in subjects with chronic neck pain have revealed reduced maximal speed in neck pain related to trauma [8,9], as well as in non-traumatic neck pain [8]. Assessment of fast cervical movements can add information on function since fast movements, more than, e.g., self-paced ROM tests, rely on muscle strength and coordination, feedforward control and self-confidence in movement ability. Hence, tests of fast cervical movements may, in addition to slower movements, be useful for characterization of neck pain disorders and for evaluation of rehabilitation. However, to our knowledge there is no data on test-retest reliability for such tests. Moreover, evaluation of whether impairments in cervical rotation speed is associated with symptoms and self-rated functioning would add information about the clinical validity of such tests.

In addition to assessing movement speed, important information about the character of sensorimotor alterations can be gained by evaluating specific features of the movement such as movement smoothness and conjunct (accessory) motions. Smooth motion is characteristic of well-coordinated muscle activity and healthy motor control [e.g., [11]]. Formally, this came to be known the "Minimum Jerk" description, initially proposed to explain central preplanning of simple hand motion [12]. Accordingly, point-to-point hand movements on a horizontal plane result in roughly straight-line paths with characteristic unimodal and symmetric "bell-shape" tangential velocity profiles [12]. Movement smoothness in cervical rotations has rarely been investigated, but reduced smoothness in chronic neck pain has been reported for slow or self-paced unconstrained movements [6,10]. When performing cervical axial rotations some conjunct movements occur also in the associated planes [13]. Reduced magnitude of conjunct movements during self-paced axial cervical rotations has been displayed in neck pain disorders, a finding attributed to altered control strategies [14].

The objective of the present study was to investigate changes in kinematics, including peak speed, movement smoothness and conjunct movements, in fast cervical rotations in women with chronic non-specific neck pain. In addition, we wanted to investigate the influence of comorbidity of low-back pain because in a parallel study we found that concurrent low-back pain was associated with increased postural sway (unpublished data). Secondary aims were to evaluate the clinical validity of the test by evaluating associations with symptoms, fear of movement and self-rated functioning as well as to estimate the test-retest reliability.

## Methods

The study had a combined cross-sectional and a test-retest reliability design. The data were derived from two separate samples, where the cervical rotation test at maximal speed was one of several sensorimotor function tests. The tests were identical but the two data sets were collected by different experimenters. Sample-1 was collected in a reliability study, including a retest after one week. Sample-2 was the pre-intervention measurement of a randomized controlled trial (ISRCTN trial registration number, ISRCTN92199001). The studies were approved by the Ethical review board in Uppsala and written consent was obtained from all participants in accordance with the declaration of Helsinki.

### Subjects

Sample-1 included 16 women with non-specific neck pain (NS) and 16 healthy women as controls (CON). Subjects were recruited by advertising in local papers and by written and verbal information to job holders in the city of Gävle, Sweden. The inclusion criteria were: woman of 20-55 years of age who reported neck pain of non-traumatic origin with a duration of at least three months from the onset of symptoms, a decreased physical functioning associated with the neck pain measured as >9 normalized points of the first 19 items in the Disability Arm Shoulder Hand (DASH) questionnaire [15]. Pain drawings were used to confirm the location of pain in the neck region [16]. Subjects with pain below the elbow were manually examined for possible cervical radiculopathy. Subjects reporting dizziness and balance disturbance were investigated for possible vestibular disorders. Subjects were excluded if the clinical examination for cervical radiculopathy or vestibular disorder was positive. Other exclusion criteria were: surgery or injury with fracture or luxation of the spine and/or shoulder, or evidence of any neurological, vestibular, psychiatric or rheumatic disease.

Sample-2 consisted of 102 NS and 33 CON subjects, women only. The same inclusion and exclusion criteria as in Sample-1 were used, except that the age range was 25-65 years and that the NS group should have indicated the neck as 'the most painful area' in a pain drawing. In addition, to the recruitment procedure used for Sample-1, invitations were sent to women with neck pain via the Social Insurance Authority, primary health-care and occupational health services. Table 1 displays descriptive data for the two samples.

**Table 1:** Descriptive data of the study samples.

	Sample-1		Sample-2	
	CON (n = 16)	NS (n = 16)	CON (n = 33)	NS (n = 102)
Age (years)	45 ± 10	48 ± 7	47 ± 10	51 ± 9*
BMI	23.8 ± 1.7	25.6 ± 4.9	24.9 ± 4.1	26.7 ± 4.7
Weight (kg)	65.7 ± 7.0	71.4 ± 14.6	70.0 ± 14.2	73.4 ± 13.7
Height (cm)	166 ± 6	166 ± 7	167 ± 7	166 ± 6
SF-36 PCS	55 ± 6	44 ± 8**	56 ± 4	41 ± 7**
SF-36 MCS	47 ± 13	41 ± 12	51.8	47 ± 7*
Physical activity	4.8 ± 0.6	3.9 ± 0.9**	4.4 ± 1.4	4.0 ± 0.9**
Duration (months)	NA	132 (53-336)	NA	120 (6-456)
VAS pain	NA	62 ± 16	NA	NA
NRS pain	NA	NA	NA	5.4 ± 1.6
NDI	NA	28 ± 13	NA	28 ± 11
TSK	NA	33 ± 13	NA	27 ± 13

Values are n, mean and ± one standard deviation, except for Duration, for which median and range values are presented. The NDI and TSK scores are normalized to the range of 0 to 100. BMI: Body Mass Index; SF-36 PCS: Short Form 36 physical component summary; SF-36 MCS: Short Form 36 mental component summary; Physical activity: How physically active at leisure time have you been in the last year? (1-6); VAS pain: Visual analogue scale rating of pain (100 mm); NRS pain: Numerical rating scale of pain (0-10); NDI: Neck Disability Index; TSK: TAMPA Scale of Kinesiophobia; NA: Not answered.

* p < 0.05, ** p < 0.01, t-test (Mann-Whitney for Physical activity) NS-CON separately for Sample-1 and Sample-2.

### Measurements

Cervical kinematic data were recorded with an electromagnetic tracking system (FASTRAK™, Polhemus Inc, USA). In this system, the location (Cartesian coordinates) and orientation (Euler angles) of small receivers are captured relative to a fixed transmitter that emits a magnetic field. The transmitter was located at waist height between the subjects' knees. Two receivers, one positioned on the forehead and the other on the dorsal spinal process of Th2, were used for recording the head rotation relative to the trunk. The sampling rate was 60 Hz. In-house software run on a PC was used to deliver pre-recorded verbal instructions and for data sampling.

The test was performed with the subject in a sitting position performing axial rotational movements of the head to the right and to the left. First, the subject was instructed to close the eyes and thereafter attain a neutral position with the head facing forward. Then the subject was instructed to turn the head "as fast as possible" to the right or left at the sound of a beep tone, and return with a self-paced speed to the neutral position. Six rotations were performed in alternating directions, with half the group starting with rotation to the right and the other half to the left. Two practice trials, one to each side, were performed prior to the test in order to familiarize the subjects with the task.

Immediately before and after the cervical axial-rotation test in Sample-2, pain was assessed with a 11-point numerical rating scale (NRS), ranging from 0, "no pain" to 10 "worst imaginable pain" [17]. This measure was included to assess possible pain induced by the test. Within one week before the testing day, pain ratings and questionnaires were completed. In Sample-1, self-rated pain was assessed as "pain at the moment" on a blank 100 mm visual analogue scale (VAS), on which 0 mm corresponds to "no pain at all" and 100 mm to "worst imaginable pain" [18]. In Sample-2, self-rated pain was assessed as "average pain during the past week" with NRS.

Identical questionnaires were completed by Sample-1 and -2. The Short Form Health Survey (SF-36) was used as a measure of general health and well-being [19]. Neck-related disability was measured using the Neck Disability Index (NDI) [20]. Upper-extremity disability was measured with DASH [15]. Fear of movement was assessed using the Tampa Scale of Kinesiophobia (TSK) [21]. Higher scores of SF-36 reflect better health status, while higher scores of NDI and DASH indicate more disability, and higher TSK scores indicate more pronounced kinesiophobia. Additional aspects of functions and symptoms that we considered to be of importance for the association analyses were addressed by separate questions (Table 2). A six-level scale was used for each additional question with alternatives corresponding to 1. Not at all/Nothing, 2. Weak/Mildly, 3. Moderate, 4. Quiet high/Somewhat strong, 5. High/Strong, 6. Almost unbearable/Maximal.

**Table 2:** Predictor variables selected for multivariate orthogonal partial least square (O-PLS) regression model for cervical kinematics

Total scores/index scores	From NDI	From DASH	Additional questions
TSK	Pain intensity	Arm, shoulder or	Age
SF-36 PF	Headache	hand pain	Symptom duration
SF-36 BP	Concentration difficulties	Tingling (pins and needles) in your arm, shoulder or hand	How physically active at leisure time have you been in the last year
SF-36 GH	Sleeping disturbance	Weakness in your arm, shoulder or hand	Can you, due to neck problems:
SF-36 VT	Car driving		Bend the head forward
SF-36 SF			Bend the head backward
SF-36 MH			Bend the head to the right
DASH 1-19			Bend the head to the left
			Turn the head to the right
			Turn the head to the left
			Run
			Do you experience:
			Jaw disorder
			Difficulty swallowing
			Clumsiness of the hands
			Dizziness
			Balance disturbance
			Sensory disturbance
			Sensitivity to light
			Sensitivity to sound
			Nausea
			Neck pain during activity
			Neck pain during rest
			Neck stiffness
			Neck tenderness
			Neck tension
			Neck fatigue
			Neck weakness
			Neck crepitations
			Neck lockings

NDI: Neck Disability Index; DASH: Disability of the Arm, Shoulder and Hand; TSK: TAMPA Scale of Kinesiophobia; SF-36 PF: Short Form-36 physical functioning; SF-36 BP: Short Form-36 bodily pain; SF-36 GH: Short Form-36 general health; SF-36 VT: Short Form-36 vitality; SF-36 SF: Short Form-36 social functioning; SF-36 MH: Short Form-36 mental health.

### Data processing

Cervical kinematics were computed using the helical axis method, which describes the movement of a segment as a rotation around and a translation along an axis that is allowed to move in space [22]. This method gives the possibility to capture a 3 D rotation in one angle signal, and also to study how the direction of the rotational axis changes during a movement. The raw data from the head and Th2 FASTRAK receivers were first low-pass filtered using a 2^nd^-order Butterworth filter and a cut-off frequency of 20 Hz. The rotation matrix of head and upper body was constructed for each time-sample from the Euler angles using the ZYX cardan sequence [13]. Head movement was calculated relative to the upper body (i.e., relative rotation matrix **M**= **M**_thorax_^T^*M_head_). The helical angle of the head relative to the upper body was then extracted from this rotation matrix [23]. In general, 3 D angular velocity cannot be obtained from direct differentiation of a set of attitude angles because angular displacements that result from matrix operations are non-commutative. The 3 D rotation velocity was therefore calculated based on Poisson equation, which takes both body position and time derivative of body position into consideration [22]. The direction vector of the finite helical axis was estimated for each time-frame using a moving window of 4 degrees [8]. This means that axis direction was computed in relation to the nearest (earlier) point in time that corresponded with a helical angular displacement ≥4 degrees. This limit was set because errors in axis calculations can become large for smaller rotations, even though the amount of rotation is well-defined [24,25].

**Figure 1:**
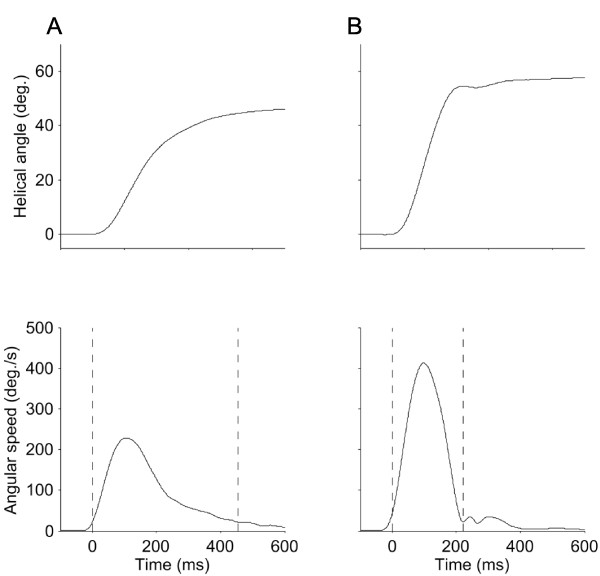
**Movement and speed profiles from a subject with neck pain and a healthy control**. Exemplar plots of the helical angle (upper panels) and the corresponding 3 D angular speed (lower panels) for the outward rotation in one trial for one subject from the neck pain group (A) and the control group (B). Both subjects have Peak Speeds comparable to their respective group means (group mean and SD for Peak Speed was 226 ± 88 for NS and 348 ± 92 for CON for the pooled samples). The dashed vertical lines represent the start and stop of the movement where a 10% value of the Peak Speed was reached.

Kinematic variables were calculated only for the outward rotation. For each trial, the helical angle and 3 D angular speed data were up-sampled to 100 Hz to increase the resolution for determination of Peak Speed and the start and stop of the movement. Peak Speed was determined as the peak rotational speed of the cervical movement. The start and stop of the movement were defined using a threshold value 10% of the Peak Speed (Figure 1). The range of the movement (ROM) was calculated as the difference in helical angle between the previously defined start and stop of the movement. The definition of start and stop of movement caused the speed profile to start and stop at non-zero levels. Therefore, the speed profile was extrapolated to zero speed using a quintic spline function that modeled the missing data based on the history of the profile. This extrapolated speed profile was used to compute movement time (Move Time = duration of the extrapolated speed profile), time to peak speed (TTP = time from start of the extrapolated speed profile to peak speed), normalized peak amplitude (NPA = Peak speed/Mean speed), acceleration-deceleration ratio (A/D-ratio = TTP/(Move Time - TTP)) and a Speed Index of Deviation (SID) from a modeled minimum-jerk speed profile. SID was used as an index of how well the speed profile could be described by optimally-smoothed speeds. SID was thus the root-mean square error (in %) calculated from the difference between the fitted minimum-jerk speed profile [12] and the extrapolated speed profile of the observed data. The amount of conjunct movements, (CM) was here defined by the change in the direction of the rotational axis during the movement. This was done by calculating the condition number for the finite helical axis direction vectors (from start to stop of movement). The condition number reflects the degree of similarity (in direction) between a collection of vectors. A relatively small condition number indicates that the change in direction of the rotational axis during the movement was relatively large, i.e. the amount of conjunct movements is large. In all, six kinematic variables were included in the analyses: Peak Speed, ROM, NPA, A/D-ratio, SID and CM. For all variables, mean values over the 6 trials were used in the analyses. Left and right rotations were pooled since evaluation of pain location from the pain drawings showed that a majority of the subjects had central or bilateral neck pain (Sample-1; n = 11 and Sample-2; n = 76).

### Statistics

SPSS for Windows 15.0 (SPSS Inc., Chicago, Illinois, USA) or SIMCA-P 11.0 (Umetrics AB, Umeå, Sweden) (for PLS-analyses only, see below) was used for statistic calculations and *P*-values below 0.05 were considered to be significant. Independent samples t-tests were used to test for group differences. Sensitivity and specificity of the kinematic variables for differentiating between NS and CON groups were evaluated by Linear Discriminant Analysis with 'leave one out' cross-validation. Stepwise modeling was used when entering all variables in the model. For kinematic variables correlated with age, residuals calculated from a linear regression with age were used in the analyses for group differences and the linear discriminant analyzes. The residuals were calculated on the pooled data for the NS and CON groups. Pain assessment with NRS immediately before and after the test in the neck pain group in Sample-2 (n = 102) was analyzed with Wilcoxon signed-ranks test.

Test-retest reliability was evaluated in Sample-1 for the NS (n = 16) and the CON (n = 16) groups separately. Relative reliability concerns the consistency of the position of the individuals, while absolute reliability apply to the consistency of the values of subjects in repeated tests [26,27]. Intraclass correlation coefficients (ICC_2.1_), a two-way random effects single-measure model (consistency) was calculated to reflect relative reliability. Since the ICC is influenced by the between subject variance (i.e., if the score variance is sufficiently large, reliability will always appear high), it is important to also present values for absolute reliability. For absolute reliability, the standard error of measurement (SEM) was calculated by dividing the standard deviation of the difference values (difference between test 1 and 2) with the square root of 2 [26]. SEM is an estimate of the expected random error, or trial to trial noise in the data. The average within-subject coefficient of variation (CV) is a measure of the relative error and was calculated using the same formula as for SEM but with the variables log-transformed [26]. CV is expressed as a percentage value (i.e., standardized value), and is applied to data in which the degree of agreement between tests depends on the magnitude of the measured values (i.e., when heteroscedasticity is present). The minimal difference (MD) was calculated as SEM × 1.96 × √2 and represents the range in which 95% of the values will be found [27]. MD is clinically useful as it represents the minimum detectable change in the unit of the measurement. To evaluate heteroscedasticity, the correlation between the average of test and retest and the absolute difference between test and retest was tested using Pearson's rank correlation test [28]. Paired t-test was used to control for bias between test occasions on the group level. Kinematic data where heteroscedasticity or skewness was present were log-transformed prior to ICC calculation. Also, kinematic variables with skewed distribution were log-transformed prior the t-tests.

To analyze associations between cervical rotation kinematics and self-rated characteristics we used orthogonal Partial Least Squares regression (O-PLS), which is a multivariate regression method. The reason for using O-PLS instead of traditional multiple linear regression analysis resides in the technique's ability to analyse many non-independent (i.e. collinear) variables. Moreover, O-PLS can handle noisy data structures, fewer observations than predictor variables and missing data [29]. An O-PLS model may reveal relationships between two blocks of variables, predictor (X) and response (Y) variables [30]. The O-PLS models are here described with the statistical parameters explained by variation (R^2^) and predicted variation (Q^2^). Q^2 ^is assessed by cross-validation and reflects the predictive ability of a model. We considered a model to be significant if the Q^2 ^> 0.05. The relative contribution of each x-variable to the PLS model (i.e., the correlation to Y and the relative importance in the X-block) is expressed as a VIP-value (variable importance in the projection). Generally, a VIP-value greater than 1 is considered as influential (significant), while values less than 0.5 indicates unimportant variables [29]. We considered VIP-values greater than 1 and with a confidence interval not including 0.5 to indicate a significant x-variable for a model. The data distributions for all variables were evaluated and log-transformed if recommended by the built-in function of the software. All analyses were performed on mean-centered and scaled data. We used the kinematic variable that best discriminated between groups as the response variable (Y). As predictors (X-variables) we used the subscales PF, SF, BP, MH, VT and GH from the SF-36 and the total scores of the TSK, as well as the sum of DASH questions 1-19, since each of these subscales/scores may be considered distinct theoretical concepts. Individual questions from NDI and symptom questions from DASH and additional questions on symptoms and physical functioning were also included. In total, 44 variables (Table 2) were entered as predictors into the model.

## Results

All participants were able to complete the test protocol. In total, 7 trials (0.7% of all trials) from 6 participants were discarded due to atypical movements. The mean pain ratings was 4.4 immediately before and 4.7 directly after the test in the NS group in Sample-2 (n = 102) (*P *< 0.01). Figure 1 shows exemplar plots of the kinematics for the outward rotation for one representative NS and CON subject, respectively.

### Test-retest reliability

Reliability statistics for cervical kinematics for the two test occasions for the NS and CON groups in Sample-1 are presented in Table 3. There was no significant bias between test occasions for any of the variables. For Peak Speed, ROM and A/D-ratio, ICCs were moderate to high, and CVs were lower compared to the other variables, indicating that these were the most reliable variables. In contrast, low ICCs and, compared to the other variables, high CVs were found for SID and CM. NPA on the other hand showed low ICC in combination with low CV for both groups. A significant difference between the groups was present for SEM of CM, evident by the fact that the CIs did not overlap between the groups.

**Table 3:** Reliability statistics of the kinematic variables (mean ± SD or 95% confidence intervals)

Variables	Group	Test 1	Test 2	ICC	SEM	MD	CV
Peak Speed (degrees/s)	CON	365 ± 96	345 ± 82	0.75 (0.41-0.91) ††	41 (31-64)	114	12.4 (9.0-19.8)
	NS	271 ± 125	253 ± 118	0.84 (0.58-0.94) ††	33 (25-52)	93	17.6 (12.7-28.6)

ROM (degrees)	CON	63.5 ± 7.7	61 ± 6.2	0.64 (0.21-86)	4.2 (3.1-6.4)	11.5	6.9 (5.1-10.9)
	NS	59.2 ± 9.1	57.6 ± 11.1	0.86 (0.63-0.95)	3.8 (2.8-5.9)	10.6	7.8 (5.7-12.4)

NPA	CON	2.32 ± 0.21	2.34 ± 0.17	0.55 (0.08-0.82)	0.12 (0.09-0.18)	0.33	5.7 (4.1-8.9)
	NS	2.31 ± 0.14	2.29 ± 0.14	0.37 (-0.16-0.73)	0.11 (0.08-0.18)	0.30	5.0 (3.7-7.9)

AD-ratio	CON	0.83 ± 0.29	0.81 ± 0.18	0.78 (0.45-0.92)	0.13 (0.10-0.20)	0.36	20.1 (18.6-73.5)
	NS	0.81 ± 0.28	0.76 ± 0.28	0.79 (0.48-0.92) ††	0.12 (0.09-0.18)	0.33	17.2 (12.4-27.8)

SID (%)	CON	13.3 ± 5.0	14.5 ± 5.3	0.55 (0.07 - 0.82)	4.1 (3.0 - 6.4)	11.4	30.9 (22.0 - 51.7) †
	NS	16.3 ± 4.2	15.8 ± 5.0	0.46 (-0.05-0.78)	3.4 (2.5-5.3)	9.4	24.2 (17.3-39.8)

CM (a.u.)	CON	12.4 ± 3.7	12.4 ± 3.8	0.38 (-0.15-0.74)	2.9 (2.2-4.6)	8.1	23.5 (16.8-38.6)
	NS	18.8 ± 7.5	15.5 ± 4.9	-0.07 (-0.54-0.45)	6.5 (4.8-10.1)	18.0	44.1 (31.0-76.1)

NS: non-specific neck pain group from sample-1 (n = 16); CON: healthy control group from sample-1 (n = 16); ROM: Range of movement; NPA: Normalized peak speed amplitude; AD-ratio: Acceleration-deceleration ratio; SID: Speed index of deviation; CM: Conjunct movements; a.u.: Arbitrary units. ICC: Intracorrelation coefficient; SEM: Standard error of measurement; MD: Minimal difference; CV: Coefficient of variation. † Heteroscedasticity. †† Skewness is present.

### Discriminative capacity and group differences

All analyses from here and onwards were performed on pooled data including all subjects from Sample-1 and -2 (NS: n = 118 and CON: n = 49). The NS group had a significantly higher age compared to CON, on average 4.5 years. Pearson's correlation analysis was therefore used to evaluate if age had an effect on the kinematic variables of the cervical rotation test. The analysis showed that ROM decreased with increased age in both CON (r = -0.61) and NS (r = -0.431) groups. Peak Speed also correlated negatively with age in CON (r = -0.36), while there was a positive correlation for NPA with age in NS (r = 0.25). We therefore controlled for age by using the residuals from the linear regression analyses, performed on the NS and CON groups together, between age and the separate variables in the analyses of group differences for Peak Speed, ROM and NPA. Group differences were revealed for all variables except for NPA and A/D-ratio (Table 4). For each separate variable we used linear discriminant analyses to evaluate their sensitivity and specificity for differentiating between NS and CON. Highest sensitivity and specificity were found for Peak Speed (Table 4).

**Table 4:** Mean and SD values, and sensitivity and specificity obtained from linear discriminant analyses

Variables	Group	Values	Sensitivity	Specificity
Peak Speed (degrees/s)	CON	348 ± 92**	74.6%	73.5%
	NS	226 ± 88		

ROM (degrees)	CON	61.5 ± 8.3**	64.4%	71.4%
	NS	52.7 ± 9.2		

NPA	CON	2.33 ± 0.20	59.3%	36.7%
	NS	2.34 ± 0.19		

AD-ratio	CON	0.81 ± 0.24	61.0%	49.0%
	NS	0.74 ± 0.22		

SID (%)	CON	13.7 ± 4.7**	58.5%	69.4%
	NS	18.1 ± 6.5		

CM (a.u.)	CON	14.5 ± 4.5**	53.4%	69.4%
	NS	17.6 ± 5.9		

Group (NS-CON) was used as grouping variable in the linear discriminant analyses, separate for each kinematic variable. NS: non-specific neck pain group, sample-1 and -2 pooled together (n = 118); CON: healthy control group, sample-1 and -2 pooled together (n = 49); ROM: Range of movement; NPA: Normalized peak speed amplitude; AD-ratio: Acceleration-deceleration ratio; SID: Speed index of deviation; CM: Conjunct movements; a.u: Arbitrary units. ** p < 0.01 (t-tests).

**Figure 2:**
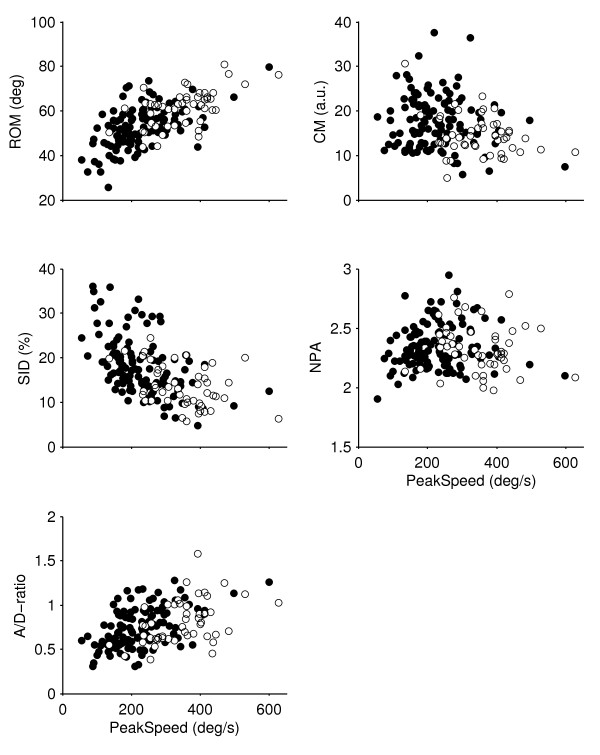
**Values of the cervical kinematic variables for the neck pain and the control subjects**. Peak Speed plotted against the other kinematic variables. Filled circles represent the neck pain subjects (n = 118) and open circles represent the controls (n = 49). ROM: Range of movement; CM: Conjunct movements; SID: Speed index of deviation from the minimum-jerk model; NPA: Normalized peak speed amplitude; A/D-ratio: Acceleration-deceleration ratio.

In Figure 2, scatter plots of the Peak Speed versus the other five kinematic variables are displayed to illustrate group differences as well as associations between variables. Table 5 provides the correlations between all kinematic variables.

**Table 5:** Pearson's correlation coefficients between all pair wise combinations of the kinematic variables

	ROM	NPA	AD-ratio	SID	CM
Peak Speed	.64**	.00	.48**	-.54**	-.25**
ROM		-.32**	.26**	-.43**	-.29**
NPA			-.20*	.27**	.00
AD-ratio				-.37**	.08
SID					.01

ROM: Range of movement; NPA: Normalized peak speed amplitude; A/D-ratio: Acceleration-deceleration ratio; SID: Speed index of deviation; CM: Conjunct movements. * p < 0.05, ** p < 0.01.

The significant correlations for most of the pair-wise combinations of the kinematic variables imply that they share common variance to a substantial extent. In order to evaluate the contribution of each kinematic variable to discriminate between NS and CON, a linear discriminant stepwise regression analysis was performed using "Group" as grouping factor. The model rendered Peak Speed (F = 51.5) and CM (F = 28.5) as the only significant classification variables. The model had a sensitivity of 76.3% and a specificity of 77.6%, of which the major part was attributed to Peak Speed. The discriminant function coefficients were 0.911 for Peak Speed and -0.321 for CM.

### Association with self-rated characteristics in neck pain

Since Peak Speed was the kinematic variable that best could discriminate between NS and CON it was used as dependent variable (Y) in the O-PLS modeling aimed at investigating association between altered cervical kinematics and self-rated characteristics in the NS group. The model explained 18% of the variance in Peak Speed and the predictive ability of the model, obtained from cross-validation, was 9.9% (R^2^Y = 0.181, Q^2 ^= 0.099). Eleven significant predictors were revealed for the NS group (Table 6).

**Table 6:** Significant variables predicting decreased Peak Speed in the neck pain group

Predictor	VIP	CI	r	R2
Car driving (NDI)	1.97	0.94	-.37**	0.14
Running	1.82	1.31	-.35**	0.12
Sleeping disturbances	1.70	0.61	-.29**	0.09
Bodily Pain (SF-36)	1.54	1.00	.26**	0.07
Arm, shoulder or hand pain (DASH)	1.52	1.16	-.25**	0.06
Bending the head to the left	1.39	0.98	-.28**	0.08
Bending the head to the right	1.38	0.98	-.25**	0.06
Pain intensity (NDI)	1.31	0.76	-.21*	0.04
Bending the head backwards	1.26	0.61	-.23*	0.05
Rotating the head to the right	1.17	0.66	-.20*	0.04
Rotating the head to the left	1.13	0.80	-.20*	0.04

Orthogonal Partial Least Squares model for Peak Speed for the neck pain group (n = 118) using self-rated characteristics as predictors. The Variable Influence on Projection (VIP), the lower limit of the confidence interval (CI) for the VIP and Pearson's correlation coefficients (r) are shown for the significant predictors, i.e., with VIP > 1 and a lower limit of VIP CI > 0.5. SF-36: short form-36; DASH: Disability of the Arm, Shoulder and Hand; NDI: Neck Disability Index. *p < 0.05, **p < 0.01.

We also investigated the effect of concurrent low-back pain on Peak Speed. Subjects in the neck pain group with concurrent low-back pain (n = 62, 209 ± 76 °/second) had a lower Peak Speed compared to neck pain subjects without low-back pain (n = 56, 245 ± 96 °/second) (*P *= 0.024, t-test). The sub-group of NS without low-back pain still displayed slower Peak Speed than CON (*P *= <0.01, t-test).

## Discussion

A significant group difference between NS and CON was found for Peak Speed, ROM, SID and CM in the fast cervical rotation test. The linear discriminant analysis using the six kinematic variables as classification variables revealed that Peak Speed and CM were significant in the model. Together, they had a sensitivity of 76.3% and a specificity of 77.8% in discriminating between NS and CON. Peak Speed was lower in NS compared to controls and even lower in the NS subjects with concurrent low-back pain compared to the NS subjects without low-back pain. Significant associations were found between Peak Speed and self-rated characteristics. Eleven significant predictors were revealed which could explain 18% of the variance in Peak Speed in the NS group, and these had a predictive ability of 9.9%. Test-retest reliability showed no systematic bias between test occasions. Further, measures of absolute and relative reliability supported the usefulness of the test on group level although the reliability varied substantially between variables. Best relative and absolute reliability were found for Peak Speed, ROM and A/D-ratio.

Peak Speed was the variable that could best discriminate between NS and CON, as evident in the results from t-tests and the linear discriminant analyses. In addition, Peak Speed was, together with CM, significant in the linear discriminant model including all kinematic variables. The lower Peak Speed in the neck pain group is coherent with findings reported in previous studies [8,9]. In contrast, Grip et al. [8] did not find any group differences in CM, which may be due to their much smaller sample. This tentative explanation is supported by the poor retest reliability we found for CM. The smaller deviation of the axis of rotation during the movement, however, is congruent with the findings of Woodhouse and Vasseljen [14] who reported less conjunct movements in associated planes in self-paced maximal range axial rotation in subjects with non-specific neck pain. In line with our data, Feipel et al [10] found differences between healthy controls and patients with cervical disc hernia or WAD for a kinematic variable that may be comparable to our SID (a mathematical model describing maximal smoothness control). In the present study, the group difference for SID could largely be explained by the significantly lower Peak Speed in NS compared to CON because as a movement is slower, it is expected to become less smooth. ROM could also discriminate between NS and CON with reasonable sensitivity and specificity. However, ROM as well as SID were significantly correlated with Peak Speed (r > 0.5, Table 5), which probably explains why they were excluded in the stepwise linear discriminant model.

An increase in pain was seen immediately after, compared to before, the test for the neck pain group in Sample-2. However, this increase was small (only 0.3 units on the NRS scale). Also, there were no drop-outs from the test and no significant difference in the kinematic variables were seen between test 1 and 2, implying that experiences from the first test occasion did not limit the performance at the second test occasion. Together, these findings support the feasibility of the test.

The relative reliability analysis demonstrated a high correlation for Peak Speed [31], and also the absolute reliability for Peak Speed supports its usefulness for evaluation purposes on group level. Interpretation of the test on individual level, however, needs caution due to the rather large standard deviation of measurements and overlap between groups. CM, on the other hand, displayed low relative reliability values and less precise estimates of absolute reliability. The questionable reliability and low sensitivity and specificity call for caution of interpretation and further evaluation of the usefulness of this variable. The negative ICC for NS subjects for CM indicates that this test is not ideal for testing conjunct motion, or that the conjunction motion is not a reliable variable. It may be possible, however, to increase the reliability by refining the measurements; for example, by increasing the number of trials or increasing measurement precision if the low reliability was due to low signal-to-noise ratio. It is also possible that assessment of CM in slow movements would yield better reliability. The relative reliability indicated by the ICC values is considered moderate to high [31,32] for all variables except NPA, SID and CM. For NPA a contributing factor was likely a small between-subject variation in the measurement values since the within-subject variation was minor (CV~5%). For SID and CM, on the other hand, the within-subject variation was high which could explain the poor ICCs for these two variables.

The clinical validity of the fast cervical rotation test was supported by the association analysis between Peak Speed and self-rated characteristics from questionnaires. The analysis revealed a significant model that could explain 18% of the variance in Peak Speed in the NS group and with a predictive ability of 9.9.%. Although this may seem to be a low prediction level, it should be taken into account that there may be a substantial between-subject variation in Peak Speed unrelated to pain or functional impairment. This normal variation is apparent from the variance seen in the healthy control group (see Figure 2). The test can, however, in combination with other tests and assessments contribute with important information about altered sensorimotor function in neck pain patients. Eleven significant predictors were revealed in the model. Three were related to pain, while five were related to difficulty in performing cervical movements. The two strongest predictors in the model were related to activities: car driving and running. Notably, car driving may involve fast cervical rotations, for example, when quickly scanning for approaching vehicles from left and right at intersections, which may explain this predictor. Running is a strenuous physical activity that involves recurring vertical movements with impacts that influence the whole body and are also likely to challenge the control of the cervical spine.

The slower movements of the NS group may be attributed to altered motor control due to pain, see e.g., [33-35] and/or mechanical dysfunction. Another explanation may be fear avoidance behavior due to the pain. However, TSK, which was included in the association analysis between Peak Speed and self-rated characteristics, did not turn out as a significant predictor in the model (VIP 0.65, the lower limit of the CI < 0.00). This speaks against fear avoidance being a main explanation for slower movements in the NS group.

A large set of predictors, involving many different bodily functions and activities, were included. The significant predictors involved items related to neck pain and functions, which may not be surprising. Nevertheless, this also supports the validity of self-ratings for evaluating specific neck functions.

The subgroup of NS subjects with concomitant low-back pain had significantly lower Peak Speed compared to the NS subjects without low-back pain. This finding supports the notion that generalized spinal pain is associated with greater disability than localized conditions [36]. The finding emphasizes the need to consider comorbidity with low-back pain when studying sensorimotor functioning in neck pain disorders.

In our experimental design, we have chosen not to standardize ROM. This may have been a limitation because if a subject could utilize a large ROM this would allow for a greater Peak Speed. This is evident from the correlation analyses (Table 5) and may have reduced the discriminative capacity of the test. Alternative test designs where an explicit target is defined, e.g., using a pointing task with a head-mounted laser, could address this problem. Such constraints may, however, alter the motor strategy [37]. Another possibility would be to define a target position by audio feedback or kinesthetic guiding. Further, the large difference in Peak Speed between NS and CON made it difficult to compare kinematic aspects influenced by the speed of rotation. Although this was most likely a limitation for the evaluation of the shape-related variables, they may still be valuable variables for longitudinal studies. Lastly, the test-retest sample consisted of 16 subjects in each group. This rather small sample size may have limited the precision of the reliability estimates [26].

## Conclusions

The results imply that cervical axial rotation speed is reduced in women with chronic non-specific neck pain, and even further reduced in subjects with concomitant low back pain. Fast cervical rotation test seems to be a reliable and valid tool for assessment of neck pain disorders and evaluation of treatment effects on group level, while caution is needed in the interpretation of individual measurements. The feasibility of the test for this patient group was supported by the fact that the subject, on average, reported only a slight increase of pain after the test. The effect of comorbidity of low-back pain in neck pain conditions on cervical movements should be considered in future research and in rehabilitation of neck pain disorders.

## Competing interests

The authors declare that they have no competing interests.

## Authors' contributions

UR conceived of the study and design, participated in the data processing and statistical analysis and drafted the manuscript. MD conceived of the study and design, carried out the data processing, participated in statistical analyses and helped to draft the manuscript. MB conceived of the study and design, participated in the data processing and helped to draft the manuscript. CH-R conceived of the study and design, participated in statistical analyses and helped to draft the manuscript. HG conceived of the study and design, participated in the data processing and helped to draft the manuscript. DGL conceived of the study and design, participated in the data processing and helped to draft the manuscript. All authors read and approved the final manuscript.

## Pre-publication history

The pre-publication history for this paper can be accessed here:

http://www.biomedcentral.com/1471-2474/11/222/prepub
